# Urinary Neutrophil Gelatinase-Associated Lipocalin (NGAL) Predicts Renal Function Decline in Patients With Glomerular Diseases

**DOI:** 10.3389/fcell.2020.00336

**Published:** 2020-05-29

**Authors:** Giuseppe Coppolino, Nicola Comi, Davide Bolignano, Gemma Patella, Alessandro Comi, Michele Provenzano, Laura Rivoli, Michele Andreucci, Giorgio Fuiano

**Affiliations:** Renal Unit, “Magna Graecia” University, Catanzaro, Italy

**Keywords:** glomerulonefritis, CKD – chronic kidney disease, prediction, urinary NGAL, renal function

## Abstract

**Objective:**

Available biomarkers for monitoring primary glomerulonephritides (GNs), often lack the ability to assess longitudinal changes and have great variability with poor sensitivity. Accruing evidence has demonstrated that Neutrophil Gelatinase-Associated Lipocalin (NGAL), holds promising capacities in predicting renal function worsening in various renal diseases. We aimed at analyzing urinary NGAL (uNGAL) levels in a cohort of individuals with biopsy-proven GNs in order to evaluate its ability to reflect the entity of renal damage and to predict disease evolution overtime.

**Methods:**

We enrolled 61 consecutive GNs patients still naïve to pathogenic therapy. uNGAL levels were measured at baseline and patients prospectively followed until the manifestation of a combined outcome of doubling of baseline serum creatinine and/or end-stage kidney disease requiring permanent dialysis support.

**Results:**

Median uNGAL levels were 107[35–312] ng/mL. At univariate and multivariate analyses an inverse correlation was found between eGFR and uNGAL levels (*p* = 0.001). Progressor subjects showed exceedingly increased baseline uNGAL values as compared with non-progressors (*p* < 0.001). Twenty-one patients (34%) reached the composite renal endpoint. Subjects with uNGAL values above the optimal, ROC-derived, cut-off of 107 ng/mL experienced a more rapid progression to the renal endpoint (*p* < 0.001; HR: 5.47; 95% CI 2.31–12.95) with a mean follow-up time to progression of 73.4 vs 83.5 months.

**Conclusion:**

In patients affected by primary glomerulonephritides, uNGAL may represent a real-time indicator of renal damage and an independent predictor of renal disease progression. Further studies on larger populations are warranted to confirm these findings.

## Introduction

The clinical management of primary glomerulonephritides (GNs) remains a major challenge for nephrologists. This particularly concerns their early identification, but also the choice of the optimal therapeutic pathway to procrastinate the evolution toward end-stage kidney disease (ESKD) (Floege and Amann, 2016). Renal biopsy represents the key-approach for diagnosis, risk stratification and therapeutic management of patients affected by GNs (Fuiano et al., 2000). However, its invasive nature prevents a routinely use, particularly to monitor disease evolution and therapy success over short time periods.

The measurement of proteinuria has been proved to be a good marker in such regard. However, it often lacks of sensitivity, even if stratified for glomerular filtration rate (Provenzano et al., 2020a), and it is far from being an early indicator of renal impairment, as it usually reflects an already evident damage of the glomerular barrier (Simeoni et al., 2016; Provenzano et al., 2020b).

Hence, the search for alternative, surrogate markers able to predict the tendency of renal disease worsening in a non-invasive and easily reproducible manner remains an important research priority in renal medicine.

Biomarkers, which have been tested so far for monitoring GNs, often lack in the ability to assess longitudinal changes and have great variability with poor sensitivity, mostly due to the influence of concurrent conditions (Bolignano and Coppolino, 2014; Caliskan and Kiryluk, 2014; Rysz et al., 2017; Coppolino et al., 2018). To overcome this important limitation, a focus on markers specifically correlated to kidney disease pathology is desirable (Wasung et al., 2015).

During the last years, accruing evidence has demonstrated that Neutrophil Gelatinase-Associated Lipocalin (NGAL), a small 25-kD protein released in blood and urine from injured renal tubular cells, holds interesting capacities in anticipating renal function worsening in patients affected by chronic kidney disease (CKD) (Bolignano et al., 2008c). Interestingly, this capacity remained evident even after adjustment for a variety of potential confounders, including the severity of renal impairment itself (Bolignano et al., 2009). Furthermore, other reports evidenced the ability of urinary NGAL (uNGAL) to predict successful response to treatment in idiopathic membranous nephropathy, a frequent form of glomerular disease (Yavas et al., 2013).

With this background in mind, we aimed at analyzing uNGAL levels in a small cohort of individuals with biopsy-proven primitive glomerulonephritides still naïve to immunosuppressive therapy, in order to evaluate its ability to reflect the entity of renal damage and to predict disease evolution overtime.

## Materials and Methods

### Patients and Baseline Data

We enrolled 61 consecutive patients with a biopsy-proven glomerulonephritis, who were referred to Renal Unit of the University-Hospital “Magna Graecia” of Catanzaro, Italy. The local Ethic Committee approved the study and all patients gave a written informed consent to participate. Inclusion criteria were the following: the presence of non-advanced renal impairment (intended as normal renal function or early renal insufficiency stages 1–3) and a stable renal function with no documented transitory or permanent doubling in serum creatinine levels over the last 6 months before starting the study. All patients were naïve to steroid or immunosuppressive therapy. Patients with active or previous history of malignancy, liver, inflammatory or infectious diseases, as well as those with altered blood glucose levels or active alterations in leucocyte count or formula, were excluded from the study. Patients’ history and drug assumption were carefully collected by interview and confirmed by checking patients’ record. Blood pressure was measured three times and the average value was recorded for analysis.

### Laboratory Measurements

Blood samples were collected in the morning after an overnight fast, together with a second urine collection of the same day. Ten milliliters of fresh urine were then immediately mixed with 1 mL of 10 mM tris buffer, pH 8.6 with 0.05% Tween 20 and 0.01% of NaN_3_ containing protease inhibitors (10 mM benzamidine, 10 mM aminocaproic acid, 20 mM ethylenediaminetetracetate and aprotinin). This mixture was centrifuged at 3000 rpm for 8 min at room temperature and then stored at −80°C until assayed.

Common laboratory parameters, including creatinine, uric acid, electrolytes, serum lipids, albumin, hemoglobin, 24-h proteinuria, fibrinogen and C-Reactive protein (CRP) were measured at baseline in all participants, according to routine standard methods. GFR was estimated by the Modification of Diet in Renal Disease formula, Eq. 7. Urinary NGAL levels were measured using an ABBOTT ARCHITECT^®^ instrument for the rapid quantitative determination of Neutrophil Gelatinase-Associated Lipocalin (NGAL) according to the manufacturer’s instructions. All specimens were often diluted to obtain the optimal density for analysis. The enzymatic reactions were quantified in an automatic microplate photometer. All measurements were made in a blinded, duplicate manner. NGAL levels were expressed as ng *per* mL. In order to minimize the potential influence of urine volume, all data analyses were checked by normalizing uNGAL for urinary creatinine.

### Follow-Up and CKD Progression Endpoint

After the baseline measurements, patients were prospectively followed until the established end of the observation period or the occurrence of CKD progression, as defined by a combined outcome of doubling of baseline serum creatinine and/or the end-stage kidney disease (ESKD) requiring permanent dialysis support. Patients were directly contacted in case they missed any appointment and at study conclusion, in order to minimize loss to follow-up.

### Statistical Analysis

Statistical analysis was performed with SPSS for Windows (version 24.0) and MedCalc (version 12.0). Estimating a 30% occurrence of the endpoint of CKD progression over 5 years in a population with non-advanced CKD and a correlation between uNGAL and the outcome of around 0.25, we computed a sample size of at least 54 participants to give approximately 80% power (alpha = 0.05, two-tailed) to reject the null hypothesis. Data were presented as mean ± SD, median (IQ range) or frequency as appropriate. Differences between groups were established by unpaired *t*-test for normally distributed values and by Kruskal-Wallis analysis followed by Dunn’s test for non-parametric values. Dichotomized values were compared using the *X*^2^ test. Pearson or Spearman correlation coefficients were employed to test correlations between variables with all non-normally distributed values being log-transformed to better approximate normal distributions. Variables incorporated into the MDRD formula for GFR estimation were excluded from analysis. Receiver Operating Characteristics (ROC) analyses were performed to estimate the area under the curve (AUC) for uNGAL and to find the best NGAL cut-off values able to identify individuals who progressed to the endpoint. Kaplan-Meier curves were employed to evaluate renal survival in subjects with urinary NGAL values above and below the optimal ROC-calculated cut-off level. Adjusted risk estimates for progression endpoint were calculated using univariate Cox proportional hazard regression analysis including all variables which resulted significantly different at baseline between “CKD-progressor” and “non-CKD progressor” subjects. Variables significantly associated with the endpoint were then tested in a multivariate Cox model. All results were considered significant if *p* value was <0.05.

## Results

### Baseline Data of the Study Cohort

Mean age of patients was 53 ± 17 yrs and 35 (57%) of them were male. Mean serum creatinine was 1.23 ± 0.6 mg/dL with a mean estimated GFR of 75.8 ± 22.1 mL/min/1.73 m^2^. Median 24 h proteinuria levels were 3.3[1.1–7.2] g/24 h. Median Urinary NGAL levels were 107 [35–312] ng/mL. According to biopsy results, IgA nephropathy was the leading glomerular disease (25 pts, 41.0%), while three (4.9%) patients had minimal change disease, 20 (32.8%) had membranous nephropathy, 12 (19.7%) had focal glomerulosclerosis and only one subject (1.6%) had a membranoproliferative disease. There was no difference in uNGAL levels across different types of glomerulonephritis’s (data not shown). Table 1 summarizes the main baseline data of the study cohort.

**TABLE 1:** Baseline data of the study population.

Parameter	All patients n:61	CKD progressors n:21 (34%)	Non-progressors n:40 (66%)	*p*
Male gender (%)	57	45	55	0.15
Age (yrs)	53 ± 17	57 ± 19	52 ± 16	**0.03**
Systolic BP (mmHg)	136 ± 20	140 ± 21	133 ± 18	**0.05**
Diastolic BP (mmHg)	77 ± 12	79 ± 12	76 ± 13	0.51
eGFR (mL/min/1.73 m^2^)	75.8 ± 22.1	51.6 ± 32.5	92.5 ± 39.8	**<0.001**
Serum Creatinine (mg/dL)	1.23 ± 0.6	1.58 ± 0.80	0.98 ± 0.35	**0.001**
Albumin (g/dL)	3.91 ± 1.20	3.9 ± 0.3	3.9 ± 0.3	0.82
Calcium (mg/dL)	9.08 ± 1.60	9.3 ± 0.6	9.0 ± 0.8	0.12
Phosphate (mg/dL)	5.00 ± 0.29	5.2 ± 1.7	4.9 ± 1.5	0.46
Hemoglobin (g/dL)	11.1 ± 1.44	11.0 ± 1.01	11.2 ± 1.36	0.58
Cholesterol (mg/dL)	228 ± 101	223 ± 60	230 ± 104	0.78
Triglycerides (mg/dL)	161 ± 109	187 ± 86	147 ± 75	0.15
C-Reactive Protein (mg/L)	3.05 [3.00–4.16]	5.01 [3.00–4.70]	3.80 [3.00–3.57]	0.29
Fibrinogen (mg/dL)	405.8 ± 109.8	417 ± 130	399 ± 98	**0.05**
Uric Acid (mg/dL)	5.90 ± 3.99	5.96 ± 1.48	5.87 ± 1.44	0.81
Proteinuria (g/24h/1.73 m^2^)	3.3 [1.1–7.2]	4.77 [1.12–5.12]	4.69 [0.68–4.32]	0.59
Urinary NGAL (ng/mL)	107 [35–312]	253 [150–432]	118 [75–318]	**0.001**
Histology (%)				0.07
Minimal change	4.9	9.5	2.5	
Membranous nephropathy	32.8	14.3	15	
Focal	19.7	23.8	37.5	
IgA	41.0	47.7	42.5	
Membranoproliferative	1.6	4.7	0	

*BP, blood pressure; eGFR, estimated glomerular filtration rate; NGAL, neutrophil gelatinase-associated lipocalin.*

### Baseline Correlates of Renal Function

At univariate analyses, fibrinogen levels were directly correlated to eGFR (*R*: 0.25; *p* = 0.05) while an inverse correlation was found between eGFR and age (*R*: −0.28; *p* = 0.05), systolic blood pressure (*R*: −0.39; *p* = 0.006), diastolic blood pressure (*R*: −0.22; *p* = 0.03) and, particularly, uNGAL levels (*R*: −0.45; *p* = 0.001). In multivariate model including all significant predictors at univariate analyses, only fibrinogen (β: 0.35; *p* = 0.005), systolic BP (β:−0.32; *p* = 0.05) and uNGAL (β:−0.48; *p* < 0.001) remained significantly associated to eGFR. The model explained about 46% of the total variance of eGFR. Supplementary Table 1 summarizes univariate and multivariate associations of baseline eGFR.

### Prospective Follow-Up and Renal Outcome

Twenty-one patients (34%) reached the composite renal endpoint over a mean follow-up of 83.1 ± 24.5 mo. There was no regression of serum creatinine to baseline levels in any of the individuals who progressed to the endpoint, therefore excluding a misleading manifestation of acute kidney injury instead of CKD progression.

The remaining 40 patients (66%) not experiencing a worsening in renal function completed the whole observational period (96 mo). At baseline, CKD-progressor subjects were significantly older and showed increased serum creatinine, systolic blood pressure and fibrinogen levels and lower eGFR values. On the contrary, they did not differ for other parameters such as gender, proteinuria, serum lipids (triglycerides and cholesterol), hemoglobin, CRP, electrolytes, uric acid, diastolic blood pressure and glomerulonephritis diagnosis. The main data and differences between patients experiencing or not-experiencing CKD progression are reported in Table 1.

### Prognostic Value of uNGAL on Renal Outcome

**FIGURE 1:**
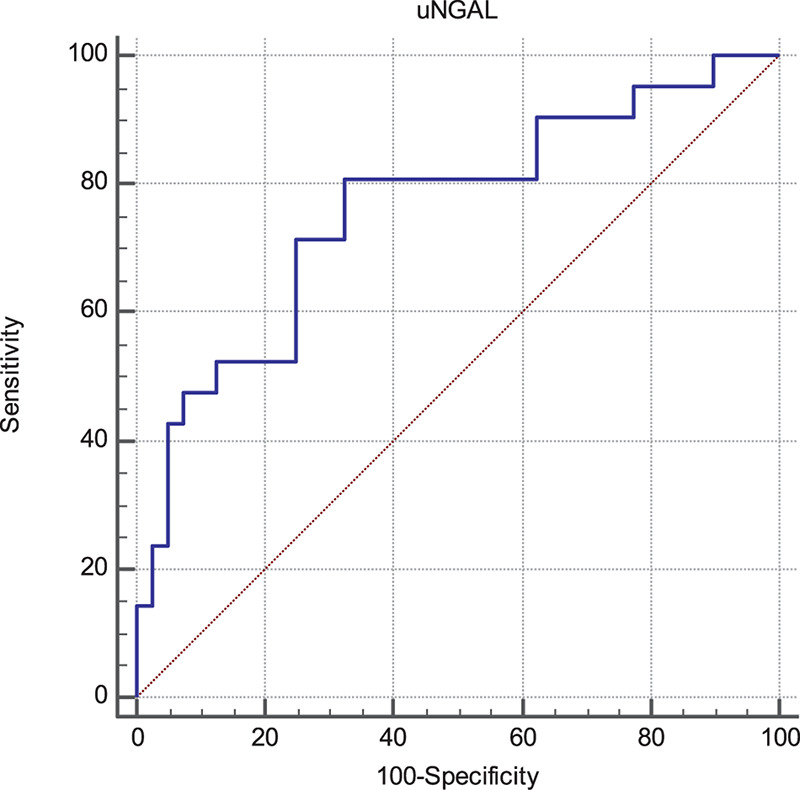
Receiver Operating Characteristics (ROC) curves of uNGAL considering progression of CKD as status variable. The Area Under the Curve (AUC) was 0.76 (95% CI 0.63–0.86). The best cut-off values able to predict the progression of CKD was found to be 107 ng/mL with a sensibility of 80.9 (95% CI 58.1–94.6) and a specificity of 67.5 (95% CI 50.9–81.4).

Progressor subjects showed exceedingly increased baseline uNGAL values as compared with non-progressors (253 [150–432] vs 118 [75–318] ng/mL; *p* < 0.001). At ROC analyses (Figure 1), the Area Under the Curve (AUC) for uNGAL to identifying progressor subjects was 0.76 (95% CI 0.63–0.86). Of note, this AUC was virtually superimposable to that of eGFR (data not shown).

The optimal uNGAL cut-off value was 107 ng/mL (Sens. 80.9%, Spec. 67.5%). Interestingly, this coincided with the median value of uNGAL in the whole cohort.

**FIGURE 2:**
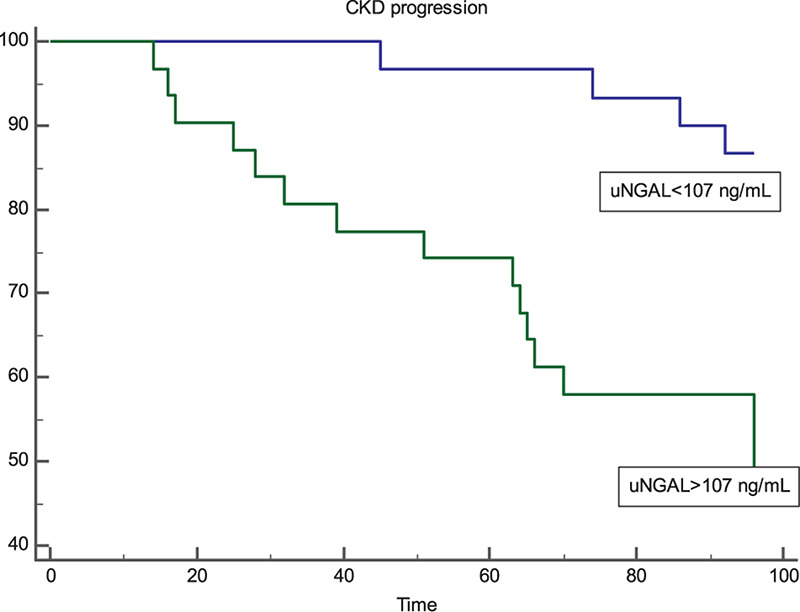
Kaplan-Meier survival curves of renal end-point in patients with uNGAL levels above and below the optimal ROC cut-off level of 107 ng/mL. Patients with uNGAL >107 ng/mL showed a significantly faster progression to endpoint (*p* < 0.001; Log-Rank Test) with a Hazard Ratio of 5.47 (95% CI 2.31–12.95).

Kaplan-Meier survival curves in individuals with uNGAL levels above or below this ROC-derived threshold are presented in Figure 2. Subjects with uNGAL values above 107 ng/mL experienced a more rapid progression to the renal endpoint (*p* < 0.001; HR: 5.47; 95% CI 2.31–12.95) with a mean follow-up time to progression of 73.4 mo (95% CI 62.8–84.6) vs 83.1 mo (95% CI 83.1–93.6).

**FIGURE 3:**
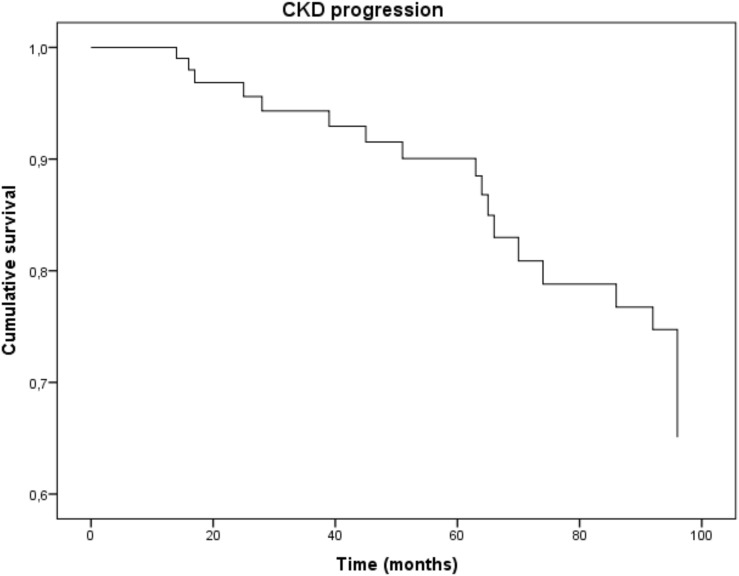
Multiple Cox-derived survival curve to CKD progression.

### Univariate/Multiple Cox Regression Analysis and Renal Outcome

To detect possible risk factors associated with the renal outcome, we tested in a Cox regression analysis all variables that were different at baseline between CKD-progressors and non-progressors (age, systolic BP, eGFR, fibrinogen and uNGAL; Supplementary Table 1). Univariate analysis showed that only age (HR 0.99; 95% CI 0.68–0.986; p = 0.05), eGFR (HR 0.97; 95% CI 0.95–0.99; *p* = 0.03) and uNGAL (HR 1.01; 95% CI 1.01–1.07; *p* = 0.004) were significantly associated to the endpoint, while systolic BP and fibrinogen failed to reach statistical significance.

However, in a multiple Cox regression model including these three variables, only eGFR and uNGAL remained significantly associated to CKD progression while age apparently lost its predictive power. More in detail, an increase of 10 ng/mL of uNGAL was associated with a 4% increased risk of CKD progression (HR 1.04; 95% CI 1.01–1.08, *p* = 0.03), whereas an increase of 10 mL/min/1.73m^2^ in eGFR reduced this risk by 3% (HR 0.97; 95% CI 0.96–0.99, *p* = 0.04). Supplementary Table 1 summarizes data from univariate and multivariate Cox-regression analyses Tables 2, 3.

**TABLE 2:** Univariate Cox proportional hazards regression model for progression of CKD.

Variable	Units of increase	*HR*	*95% CI*	*X*^2^	*p*
**Age**	**1 year**	**0.99**	**0.98–0.99**	**1.22**	**0.05**
Systolic BP	10 mm/Hg	0.99	0.97–1.02	0.17	0.80
**eGFR**	**10 mL/min/1.73 m^2^**	**0.97**	**0.95–0.99**	**4.47**	**0.03**
Fibrinogen	10 mg/dL	1.00	0.99–1.00	0.55	0.45
**uNGAL**	**10 ng/mL**	**1.03**	**1.01–1.07**	**2.62**	**0.04**

*BP, blood pressure; eGFR, estimated glomerular filtration rate; uNGAL, urinary NGAL.*

**TABLE 3:** Multivariate Cox proportional hazards regression model for progression of CKD.

Variable	Units of increase	*HR*	*95% CI*	*X*^2^	*p*
Age	1 year	0.98	0.95–1.01	2.13	0.14
**eGFR**	**10 mL/min/1.73 m^2^**	**0.97**	**0.96–0.99**	**5.02**	**0.04**
**uNGAL**	**10 ng/mL**	**1.04**	**1.01–1.08**	**2.13**	**0.03**

*eGFR, estimated glomerular filtration rate; uNGAL, urinary NGAL.*

## Discussion

The main aim of our study was to test the ability of urinary NGAL to predict progression of renal disease over a long follow-up period in a selected cohort of patients affected by primitive glomerulonephritides. Indeed, in this regard, we found a significant diagnostic capacity of NGAL which was independent from other important confounders, such as the type of glomerular disease and the baseline GFR itself. After diagnosis, besides the evaluation of proteinuria and GFR slopes overtime, only invasive procedures like renal biopsy are available to follow the natural history of GNs but routinely execution of biopsy is risky and unfeasible in common clinical practice. Hence, the search for early, non-invasive, alternative biomarkers able to stratify the risk to ESKD in this particular population setting remains a demanding priority. Similarly, the ability of these biomarkers to predict risk of relapses and response to different therapies would deserve targeted explorations. Unfortunately, the present study was not sufficiently powered to analyze also this aspect due to the relatively small cohort and the large variability of patients’ diagnosis and characteristics.

In two recent studies, Bennett et al. (2012, 2017) demonstrated the capacity of NGAL to predict the degree of response to steroid therapy in children with idiopathic nephrotic syndrome, allowing to discriminate between steroid-sensitive and steroid-resistant individuals.

In our cohort, the majority of participants was affected by IgA nephropathy (41%). Hence, our findings were mostly in accordance with Ding et al. (2007), who also demonstrated that uNGAL is a powerful predictor of disease progression in patients with IgA nephropathy with tubulointerstitial injury. In contrast with these results, Neuhaus et al. (2018) failed to predict the progression of disease with a panel of urinary biomarkers including NGAL in a sub-cohort of the STOP-IgAN trial, although their first purpose was to identify subjects who could benefit from immunosuppression as compared to a supportive therapy alone. An et al. (2019) found higher urinary NGAL levels in patients with nephrotic range proteinuria as compared to those with sub-nephrotic range proteinuria. In our study, we did not find any correlation between uNGAL and proteinuria, perhaps due to the relatively limited sample size and the high heterogeneity of case-mix of our cohort. This also apparently contradicts findings from another previous paper, in which increased urinary NGAL levels resulted highly correlated with proteinuria in a larger cohort of proteinuric patients (Bolignano et al., 2008b). According to current knowledge, high urinary NGAL found in renal diseases is not merely an expression of circulating serum NGAL that is passively loss through the damaged glomerular membrane, but also the active response of tubular cells to a non-specific damage. From this point of view, in our study, the elevated levels of urinary NGAL would therefore represent a “real-time” indicator of how much damage and active suffering is present within the chronic renal impairment (Mori and Nakao, 2007; Bolignano et al., 2008a; Kuwabara et al., 2009). The measurement of urinary NGAL was demonstrated to be an early indicator of renal damage that anticipate common markers used in clinical practice like serum creatinine or cystatin C (George and Gounden, 2019). These common markers are practical and economically sustainable, but far from being a gold standard in terms of prognostic capacity. In fact, their implementation in determined algorithms allows the stadial categorization of renal impairment progression, but has the limit to hide the first phases of organ dysfunction. In fact, until more of the 50% of renal structures are destroyed, the estimated renal function may be normal or paradoxically increased due to hyperfiltration of the remnants glomeruli (Hostetter, 1995). Stressful activation of this functional compensatory effort is itself at the basis of pathological development of lesions, leading to accelerated worsening of renal function (Kliem et al., 1996). Our study has some strengths and limits that deserve mentioning. The main strength was the very long follow-up and the robust statistical analysis implant in which urinary NGAL remained an independent predictor of CKD progression even in a Cox proportional hazards model adjustment. Furthermore, all patients were still naïve to targeted drug approaches, therefore limiting the potential influence of pre-existing immunosuppressive therapy. Main limits are probably represented by the small, single-center evaluation and by the heterogeneity of GNs, which could prevent application of findings to specific GN subpopulations.

## Conclusion

We demonstrated that urinary NGAL represents a real-time indicator of renal damage and an independent predictor of renal disease progression in patients affected by primary glomerulonephritides. Future studies would be desirable to ascertain whether this biomarker could also be useful to guide therapeutic management of patients affected by GNs.

## Data Availability Statement

The datasets generated for this study are available on request to the corresponding author.

## Ethics Statement

The studies involving human participants were reviewed and approved by University Magna Graecia of Catanzaro. The patients/participants provided their written informed consent to participate in this study.

## Author Contributions

GC, GF, NC, MA, and DB contributed to the research idea. DB and GC wrote the manuscript. GP, AC, LR, and MP collected the data.

## Conflict of Interest

The authors declare that the research was conducted in the absence of any commercial or financial relationships that could be construed as a potential conflict of interest.
