# Supplementary material for: Urinary Neutrophil Gelatinase-Associated Lipocalin (NGAL) Predicts Renal Function Decline in Patients With Glomerular Diseases
Source: Front Cell Dev Biol. 2020 May 29;8:336. doi: 10.3389/fcell.2020.00336 (PMC7272710; doi:10.3389/fcell.2020.00336)
Supplement: Supplementary file 1 [file Table_1.DOCX]

**Supplementary Table 1.** Univariate and multiple regression analysis of estimated GFR at baseline.

|  | ***Partial R*** | ***β*** | ***P*** |
| --- | --- | --- | --- |
| Log uNGAL | -0.45 (p=0.001) | **-0.48** | **<0.001** |
| Systolic Blood Pressure | -0.39 (p=0.006) | **-0.32** | **0.05** |
| Diastolic Blood Pressure | -0.22 (p=0.03) | 0.43 | 0.66 |
| Fibrinogen | 0.25 (p=0.05) | **0.35** | **0.005** |
| Age | -0.28 (p=0.05) | -0.39 | 0.69 |

Multiple R=0.67, R^2^=46%; p<0.001. **β** ,standardized coefficient of correlation; **uNGAL**, urinary NGAL
